# Nanofibril Cellulose–Carrageenan Aerogel Production and the Effect of Crosslinker Addition on Aerogel Performance

**DOI:** 10.3390/polym18202464

**Published:** 2026-10-09

**Authors:** Esma Ege, Orçun Çağlar Kurtuluş

**Affiliations:** 1 Department of Biomedical Device Technology, Tosya Vocational School, Kastamonu University, Kastamonu 37000, Türkiye; egeesma2165@gmail.com; 2 Department of Materials and Material Processing Technology, Tosya Vocational School, Kastamonu University, Kastamonu 37000, Türkiye

**Keywords:** nanofibrillated cellulose, carrageenan, aerogel, tannic acid, polymer, material

## Abstract

Nanofibrillated cellulose (NFC) and carrageenan (CG) have emerged as sustainable biological polymers of plant and animal origin, and have been produced as films, spheres, and other materials with various physical macromorphological structures. However, to the best of the authors’ knowledge, NFC/CG aerogels combining TEMPO-oxidized NFC, CG, and a natural crosslinker (TA) have not been systematically characterized yet, which is the main objective of this study. NFC/CG aerogels were obtained by combining these two polymers at various mixing ratios. Samples were produced as pure aerogels without crosslinking chemicals and with tannic acid (TA) as a crosslinker. Material performance was evaluated using density–porosity, BET (Brunauer–Emmett–Teller) surface area, liquid absorption (water, phosphate-buffered saline (PBS), oil, methylene blue (MB), and Congo red (CR) penetration ratio), water vapor transmission (WVTR), chemical structure changes (FTIR), degree of crystallinity (XRD), antibacterial properties, and SEM (scanning electron microscopy) imaging tests. All aerogels exhibit densities of 0.0154–0.0267 g/cm^3^, porosities of 97.91–99.63%, and specific surface areas of 128–303 m^2^/g. In addition to bacterial resistance, aerogels had high water and PBS absorption values. The incorporation of TA led to a notable enhancement in antibacterial efficacy, though with a minor decrease in the porosity and surface area parameters.

## 1. Introduction

In light of the detrimental environmental effects and elevated global pollution levels attributed to synthetic polymers and their precursors, there has been a growing trend in recent years to substitute them with bio-based materials that do not present health hazards or environmental harm. The materials and composites sectors are concurrently concentrating on manufacturing lightweight and low-density materials with high porosity. Consequently, the investigation and application of materials derived from bio-based polymers, emphasizing sustainability, have gained prominence in recent years. Cellulose and CG have recently emerged as prominent polymers.

NFC is a biopolymer produced using a variety of mechanical, chemical, and enzymatic methods from cellulose, a component of a bio-based, renewable, environmentally friendly, and sustainable production ecosystem. It was first developed by Turbak et al. (1983) and Herrick et al. (1983) [1,2]. NFC manufacture is based on subjecting the cellulosic component to mechanical fibrillation, which degrades the woody cell wall. NFC fibers with amorphous and crystalline regions are produced by the mechanical defibrillation process; their dimensional characteristics (5–50 nm diameter, a few micrometers in length) vary based on a number of factors, including the raw material used, the pre-treatment procedure used, and the mechanical defibrillation procedure used [3,4,5]. NFC has been used in multiple application domains, including in packaging [6,7,8,9], biomedical fields [10,11,12], paper manufacturing [13,14,15], and as rheology modifiers in food and cosmetic industries [16,17,18,19], owing to its exceptional characteristics such as a high aspect ratio, crystallinity, and Young’s modulus [20].

CG, a linear, water-soluble carbohydrate, is a natural carbohydrate obtained by extracting various red seaweeds from the *Rhodophyceae* family, which are often found in the Atlantic Ocean near the British coast, Europe, and North America. Its chemical structure is sulfated polygalactane with a 15–40% ester sulfate concentration. CG is made up of D-galactose and 3,6-anhydro-galactose units connected by α-1,3 and β-1,4 glycosidic linkages. Its molecular weight typically exceeds 100 kDa. The most essential elements influencing CG’s qualities are the quantity and position of ester groups, as well as the 3,6-anhydrogalactose content in its structure. The anionic ester sulfate groups are responsible for the chemical substance’s basic reactivity. CG is classified into three categories based on the quantity of ester sulfate groups and 3,6-anhydrogalactose concentration. These are Iota (τ) (ester sulfate content 28–30%), Kappa (κ) (33%), and Lambda (41%). A higher amount of ester sulfate groups permits CG to dissolve at lower temperatures, but it decreases gel stability. The intended usage of CG is determined by its rheological characteristics. It is most often used in the food sector as a gelling agent, for thickening, as a stabilizer, and as an emulsifier, with codes E 407 and E 407a [21,22,23]. In addition to food production, CG is used as an additive in the manufacture of air freshener gels, toothpastes, flame-retardant foams, shampoos, cosmetic creams, and shoe polishes. However, in recent years it has gained popularity in pharmaceutical formulations, the cosmetics industry, and biomedical applications [21].

In various industrial applications, lightweight, porous materials with strong mechanical properties are becoming increasingly prevalent as a result of the rapid advancements and changes in polymer technology and final material production processes. Aerogels have become more significant in materials research and industrial applications owing to their distinctive characteristics, including their low density, lightweight quality, high porosity, large surface area, and superior mechanical qualities. Aerogels, produced by extracting the solvent from the gel phase using various processes (traditional drying, freeze-drying, supercritical drying, etc.), were initially discovered by Kistler in the 1930s [24]. Aerogels, which can be classed in multiple ways, are primarily characterized based on their structural components. They are categorized into three primary types: inorganic, organic, and hybrid [25,26]. In the category of organic aerogels, involving both synthetic polymer-based and biological polymer-based systems, biopolymer materials have gained significant attention in recent years and have been examined across various domains [27,28,29], including in food packaging [30,31,32,33,34,35,36,37] and biomedical applications [38,39,40,41,42,43].

Composite structures formed with the addition of cellulose and its diverse precursor molecules (nanofibril cellulose, nanocrystalline cellulose, bacterial cellulose, carboxymethyl cellulose, hydroxyethyl cellulose, etc.) are found in the forms of films [25,44,45], cryogels [46], and aerogels [47], as indicated in the research. Moreover, the efficacy of NFC/CG-based composites fabricated in different forms, including films [48,49,50,51,52,53,54], aerogels [55] and hydrogels [56], has been analyzed across a number of fields, covering food applications [25,45,50,52,54,57], water purification approaches [58], and biomedical uses [59]. In addition, different types of polysaccharide-based aerogels, including cellulose and other polysaccharides, have been reported. Rege et al. (2018) [60] studied the preparation and characterization of pectin, cellulose, and κ-carrageenan-based aerogels and established correlations between synthesis parameters and morphological entities. However, they did not use TEMPO-mediated cellulose oxidation nor a natural polyphenolic crosslinker such as TA. NFC/CG combinations have also been reported in hydrogel form, e.g., as TEMPO-oxidized systems [61] and reinforced films [62]. Composite aerogels of carboxylated polysaccharides and carrageenan [47] and TEMPO-oxidized cellulose nanofibers and other polysaccharide derivatives [63] have been studied for environmental remediation applications. However, to the best of the authors’ knowledge, a systematic characterization of aerogels combining TEMPO-oxidized NFC with CG at different mixing ratios, with and without TA as a natural crosslinking agent, has not been reported before.

The objective of this study is not to introduce a new biopolymer system, but to systematically evaluate the composition–structure–property relationship of a specific, but unexplored, biopolymer combination: (i) TEMPO-oxidized NFC with surface carboxylate groups, (ii) CG at different mixing ratios, and (iii) TA as a natural polyphenolic crosslinker. In the present study, the production and characterization of NFC/CG-based aerogels were investigated by a comprehensive set of tests, including density–porosity, BET surface area, liquid absorption, WVTR, chemical structure, crystallinity, antibacterial, and micromorphological analyses.

## 2. Materials and Methods

### 2.1. Materials

Mg-bisulfite pulp (MBP) (DP: 3113; α-cellulose: 92.5%; β-cellulose: 5.3%; Ɣ-cellulose: 1.6%) was purchased from Vian Paskov in Paskov-Czechia. Other chemicals, including magnesium chloride (MgCl_2_∙6H_2_O), monosodium phosphate (NaH_2_PO_4_), potassium dihydrogen phosphate (KH_2_PO_4_), sodium chloride (NaCl), and TA were purchased from TEKKİM Kimya San. ve Tic. Ltd., Şti., Bursa, Türkiye. Additionally, Kimbiotek Kimyevi Mad. San. (İzmir-Türkiye) provided MB, CR, dyes utilized in dye penetration tests, and other basic polymer CG (purity: 99.8%; mol. weight: 551.8 g/mol; density: ~0.8 g/cm^3^). All procedures involved the use of deionized water.

### 2.2. Methods

#### 2.2.1. Producing Nanocellulose and Determining Carboxyl Fraction of NFC

NFC was produced by using the pre-treatment of the TEMPO oxidation process before mechanical disintegration of the treated pulp. MBP was treated with 0.1 mol of TEMPO and 1 mol of NaBr, with the pH of the cellulose suspension and substances set to 10. A total of 3 mmol NaClO (pH: 10) solution was added into the slurry and mixed at 500 rpm. The pH was kept stable throughout the reaction, and, after pH stability was reached, the reaction was stopped by adding 50 mL ethanol [64]. Modified cellulose was washed with a large amount of water and passed from the high-pressure homogenizator (APV 1000, Hemisan, İzmir-Türkiye) at a concentration of 1%. Obtained NFC was stored at +4 °C before the aerogel production process.

Determination of the carboxyl fraction of NFC was carried out according to Saito et al. (2007) [65]. A 0.05 g NFC sample was dissolved in 250 mL water, and 15 mL of 0.01 M HCl was added into the suspension and stirred for 10 min. The suspension was titrated by 0.01 M NaOH, and the carboxyl fraction was calculated using the formula below:

(1) $$\mathrm{Carboxyl Amount}\;(\mathrm{mmol}/g)=\frac{V\times N}{w},$$

where V is the consumed NaOH amount during the titration (mL), N is the concentration of NaOH (mol/L), and w is the dry weight of the sample (g).

#### 2.2.2. Preparing CG, TA, and PBS Solutions

Both CG polymer and crosslinker TA solutions were prepared at 1% (*w*/*w*) concentration. PBS solution was prepared by using different salts (0.2 M NaH_2_PO_4_, 0.2 M KH_2_PO_4_, and 0.15 M NaCl), and the pH (7.4) of the solution was controlled before the tests.

#### 2.2.3. Aerogel Processes and Tests

Biopolymers of NFC and CG were prepared at 1% concentration and mixed at 75–25% (75% NFC and 25% CG), 50–50%, and 25–75%. The CG polymer was added to the NFC suspension, and the mixture was agitated at 300 rpm for 60 min using a magnetic stirrer. The mixtures were poured into glass Petri dishes, and cooled to −86 °C. Frozen gels were lyophilized at −55 °C, −50 mbar vacuum medium, for 48 h.

#### 2.2.4. Density–Porosity Measurements

A basic and common method was used to determine the density of the aerogels. Weights of the aerogels were divided to volume simply and density values were calculated as g/cm^3^. Porosity measurements were performed by a helium pycnometer (Quantachrome Ultrapyc 1200 E, Quantachrome Instruments, Boynton Beach, FL, USA), and values were calculated according to the equation given below:

(2) $$\mathrm{Porosity}\;(\%)=\frac{\left[V1-V2\right]}{V2}\times \;100,$$

where V1 is the measured density of aerogels (cm^3^), and V2 are the density values measured by the helium pycnometer (cm^3^).

#### 2.2.5. Liquid Absorption and Dye Penetration Performances

Water, PBS, and oil absorption performances were carried out to observe the absorption behavior of aerogels versus different types of liquid. For this reason, aerogel samples were placed into liquids (water: 50 mL, PBS: 10 mL, oil: 10 mL) for 24 h and, after the end of the period, the excess was removed by coarse filter paper and the sample weights were measured. Oil and PBS absorption experiments were conducted at ambient temperature, whilst the water absorption test was executed at 37 °C. Absorption rates were calculated according to the equation below. The absorption tests were conducted in triplicate (n = 3), and the results are presented as mean values.

(3) $$\mathrm{Liquid Absorption}\left(\%\right)=\frac{\left[\mathrm{Mt}-\mathrm{Mo}\right]}{\mathrm{Mo}}\times \;100,$$

where Mt is the weight of the sample after the test (g), and Mo is the sample weight before the test (g).

Dye penetration tests were conducted in three replicates for every aerogel. For this purpose, three unique samples were chosen from the aerogels generated in each mixture, and the method was performed on the most appropriate surface areas. A single droplet of dye (MB or CR) was dispensed onto the sample under ambient conditions utilizing a burette with a stopcock, and the degree of dye penetration within the aerogel was monitored after 30 s. The rate of dye penetration was calculated utilizing the following formula:

(4) $$\mathrm{Dye}\;\mathrm{Penetration}\;\mathrm{Ratio}\left(\%\right)=\frac{\left[L1\right]}{\left[L2\right]}\times 100,$$

where L1 is the penetrated dye length through the cross-section (mm), and L2 is the total length of the cross-section of the aerogel (mm).

#### 2.2.6. Specific Surface Area

The specific surface area of the aerogels was determined by using a Quantachrome Nova Touch Lx4 (Quantachrome Instruments, Boynton Beach, FL, USA) device. After applying a pressure-drying process to remove the water in the aerogels, adsorption–desorption curves were obtained and BET (Brunauer–Emmett–Teller) specific surface area values were measured.

#### 2.2.7. Water Vapor Transmission (WVTR)

WVTR tests were performed according to ASTM (E96-90 Procedure D) [66], with minor modifications. Aerogel samples were tightly stuck onto glass bottles containing 6 mL water, and parts of the samples were coated with paraffin to remove vapor diffusion. Samples were put into a desiccator (40% humidity medium), including saturated MgCl_2_∙6H_2_O. All samples were kept in an autoclave at 37 °C for 24 h. The WVTR was calculated according to equation below:

(5) $$\mathrm{Water Vapor Transmission}=\frac{\left(G/t\right)}{A},$$

where G/t is the slope of the weight change versus time (g/h), and A is the test area (m^2^).

#### 2.2.8. X-Ray Diffraction Analysis (XRD)

X-ray diffraction (XRD) analysis was performed to evaluate the cellulose-related crystalline structure of the aerogels. Cylindrical samples with a diameter of 10 mm were prepared by compressing the aerogels using a 5-ton hydraulic press for 1 min to obtain specimens with a relatively uniform geometry. XRD measurements were conducted using Cu Kα radiation (λ = 0.154 nm) over a 2θ range of 3–90° at a scanning rate of 2°/min. The Segal crystallinity index (CI) [67] was used as an empirical and comparative parameter to evaluate changes in the cellulose I-related crystalline structure among the samples. The method was originally developed for cellulose, and has subsequently been applied to cellulose nanofibrils, including TEMPO-oxidized cellulose nanofibrils. Accordingly, the CI values obtained in this study should be interpreted as relative/apparent cellulose crystallinity indices rather than absolute crystallinity fractions of the NFC/CG composite. The Segal index was calculated as:

(6) $$\mathrm{CI}\left(\%\right)=\frac{\left[I002-\mathrm{Iam}\right]}{I002}\times \;100,$$

where I_002_ is the maximum intensity of the principal cellulose I diffraction peak, corresponding predominantly to the (002) reflection at approximately 22–23° 2θ, and I_am_ is the intensity of the minimum between the principal cellulose I diffraction peaks, conventionally taken at approximately 18° 2θ. Because the aerogels contain both TEMPO-oxidized NFC and CG, the calculated values are used primarily to compare relative changes in the cellulose-related ordered structure among the investigated formulations.

Peak deconvolution was performed on the original diffraction data in the 2θ range of 10–45° without smoothing or additional background correction. Each pattern was fitted by bounded nonlinear least squares with Gaussian components for the cellulose Iβ reflections [(110), (110), (102)/(012), (200) and (004)], a broad non-crystalline halo and, for the CG-containing samples, the KCl reflections. The crystallinity index from deconvolution (CI-d) was calculated as the ratio of the summed areas of the cellulose Iβ peaks to the total area of the cellulose and non-crystalline components.

#### 2.2.9. FTIR Measurements

Measurements using FTIR spectroscopy were carried out in order to ascertain the evolution of the chemical structures of polymer-based aerogels with varying mixing ratios and without the addition of any additives. For this aim, a Bruker Alpha II instrument (Billerica, MA, USA) was employed, samples were obtained from the specimens’ upper surfaces, and measurements were performed in the 400–4000 cm^−1^ spectral region with a resolution of 2 cm^−1^. Band heights were measured on absorbance spectra (A = −log T) using a two-point linear baseline. The lateral order index (LOI = A1430/A897) and the total crystallinity index (TCI = A1372/A2900) were calculated. For the NFC-CG aerogels, the ratios were also calculated after subtracting the spectrum of pure CG scaled on the CG-specific band at 845 cm^−1^.

#### 2.2.10. Performance of Bacterial Resistance

Aerogel antibacterial activity was assessed using the disk diffusion method. Bacteria cultivated in SS (*E. coli*)- and BHA (*S. aureus*)-enriched media were disseminated on agar media, and aerogel samples with a diameter of 1 cm were placed in contact with the bacterial medium. The culture media were incubated at 28 °C for 24 h, after which inhibition zones were measured at various times and averaged.

#### 2.2.11. Micromorphological Analysis

Scanning electron microscopy was used to analyze the aerogels’ surface and cross-sectional micromorphological features (Thermo Fisher Scientific, FEI Quanta FEG 250, Hillsboro, OR, USA, accelerated voltage 11 kV). Before measurement, the samples were coated with gold-palladium at a current of 40 mA and a pressure of 50 mbar.

## 3. Results and Discussion

### 3.1. Density–Porosity and Specific Surface Area

**Figure 9:**
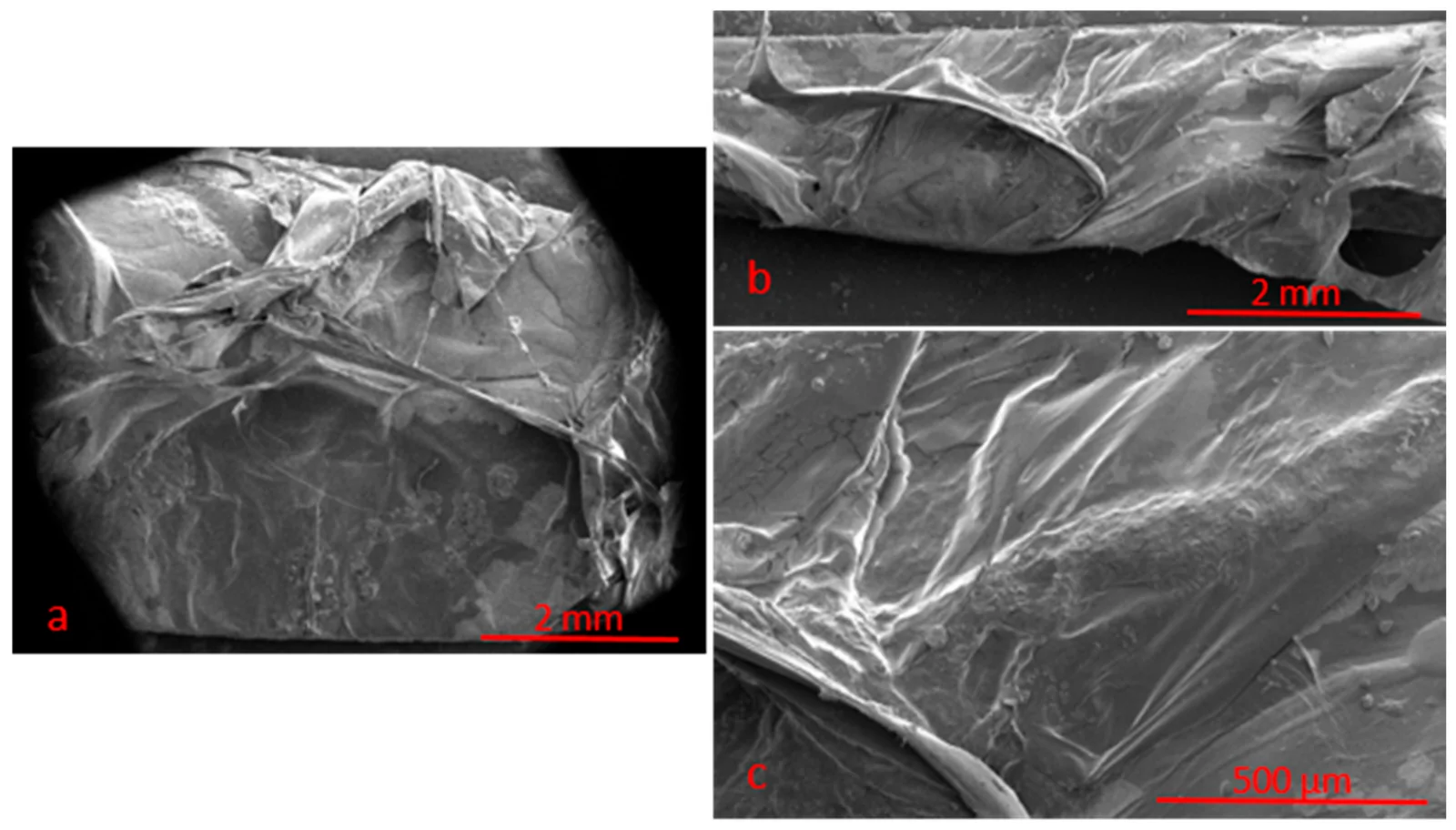
SEM images NFC-CG 50/50/5C: ((**a**) surface; (**b**,**c**) cross-section).

Table 1 shows the density, porosity, and surface area values of NFC and NFC-CG aerogels. The density values were discovered to be 0.0223 g/cm^3^ for 100% NFC and 0.0236 g/cm^3^ for pure CG. The table shows that adding CG to the NFC structure resulted in decreased density values compared to 100% NFC. Aerogels with 5% TA additive demonstrated significantly greater density values than composites with 1% TA additive. All aerogels exhibited a highly porous structure, as observed by porosity measurements. The addition of crosslinking agents led to a small drop in porosity values in the composition. The greatest porosity value was found to be 99.63% for 100% NFC, while the lowest was 97.91% for NFC-CG 25/75/5C. This phenomenon is attributed to the incorporation of TA, which facilitates closer interactions among the polymeric components, resulting in the polymer blocks being positioned nearer to one another in the structural morphology and subsequently diminishing porosity. The SEM images distinctly illustrate that the incorporation of 5% TA results in a more tightly compact and dense morphological structure in both the surface and cross-sectional morphologies of the aerogels (Figure 9a–c).

**Figure 1:**
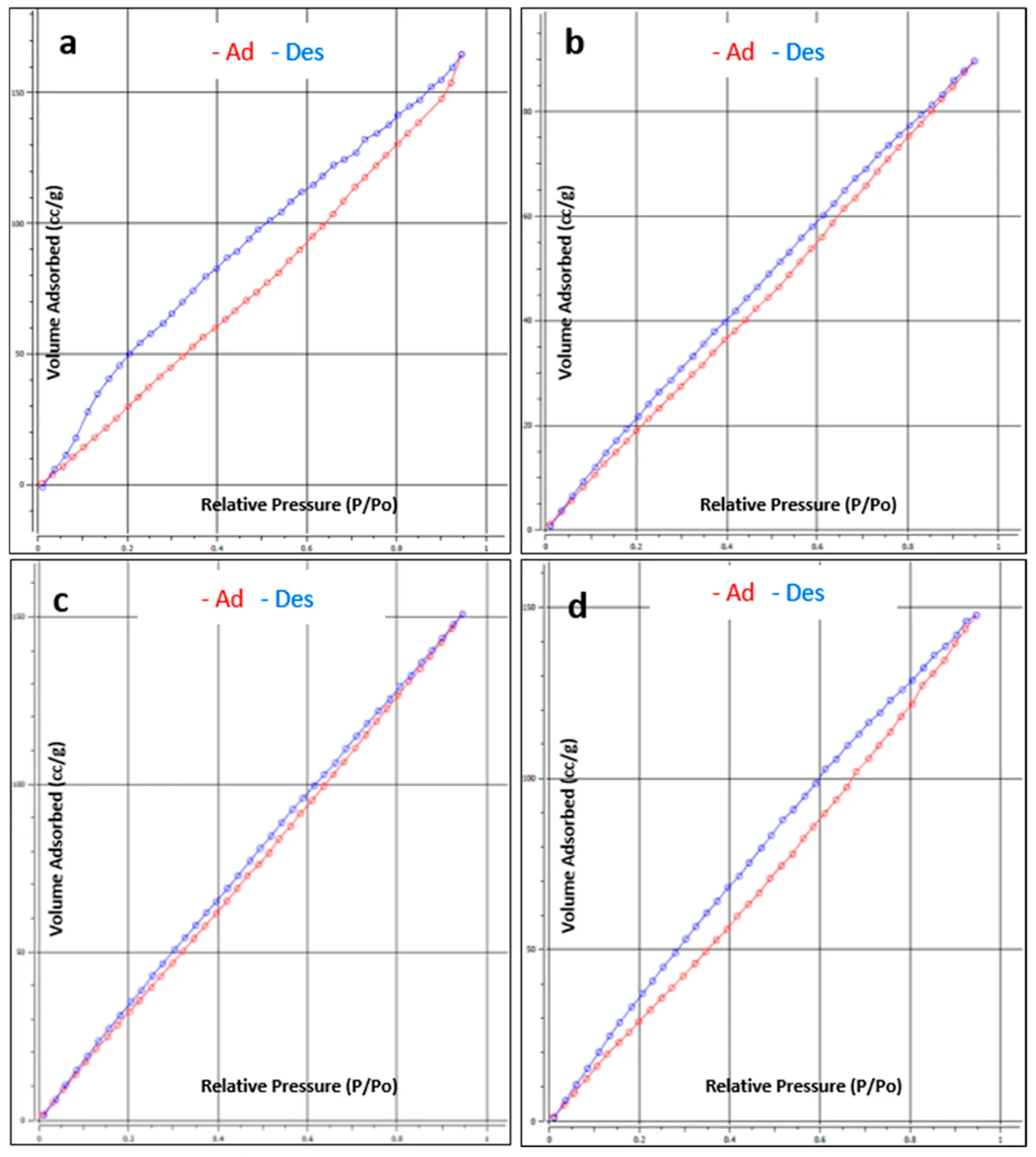
Adosption-desorption isoterms of different samples. (**a**) Pure NFC aerogel. (**b**) Pure CG aerogel. (**c**) NFC-CG 50/50-based aerogel. (**d**) NFC-CG 50/50/5C-based aerogel.

The specific surface area is an essential characteristic of porous materials like aerogels and foams. Table 1 presents surface area values, and Figure 1 shows the adsorption–desorption isotherms of pure NFC, pure CG, NFC-CG 50/50-, and NFC-CG 50/50/5C-based aerogels. The maximum surface area was seen in 100% NFC-based aerogels at 303.12 m^2^/g, whilst the minimum surface area was recorded in pure CG-based aerogels at 128.25 m^2^/g. Upon examining the surface area values of all aerogels, it was seen that, consistent with porosity values, the surface area values diminished with the incorporation of CG into the NFC structure. Moreover, an increase in crosslinker additive resulted in reduced surface area values compared to aerogels synthesized without the crosslinker additive. The reduction in the surface area and porosity of the aerogels with increasing addition of CG into the NFC structure is attributed to two essential factors. The initial step entails the incorporation of CG into the NFC structure, thereby saturating the pores within the network with CG. The second mechanism is believed to cause the pore walls to converge, resulting in a more compact and denser aerogel structure, hence leading to a reduction in specific surface area values. Rege et al. (2018) [60] synthesized polysaccharide-based aerogels from pectin, cellulose, and K-CG, and evaluated their performance. Upon examining the specific surface area and porosity values of aerogels, it was found that cellulose-based aerogels produced with a 3% polymer concentration had a porosity of 90%, whereas CG-based aerogels demonstrated a porosity of 91%. The specific surface area values revealed that cellulose-based aerogels exhibited a surface area of 491 m^2^/g, while CG-based aerogels had a surface area of 230 m^2^/g. The findings elucidate the trend of diminishing specific surface area in NFC/CG-based aerogels with the incorporation of CG.

Table 1 indicates that the incorporation of crosslinker TA into the NFC-CG matrix resulted in reduced porosity and specific surface area values compared to aerogels devoid of crosslinker additives. This phenomenon is mostly attributed to the hydrogen bonds formed between TA, characterized by its polyphenolic structure, and the -OH groups present in both NFC and CG, as well as the -SO3^−^ groups within the CG structure. The electrostatic interaction between the polymers constituting the structure and TA, which enhances the quantity of hydrogen bonds, resulted in the proximity of NFC and CG chains inside the polymeric matrix, thereby increasing crosslinking and subsequently reducing porosity and specific surface area values.

### 3.2. Absorption of Water, PBS, and Oil

**Figure 2:**
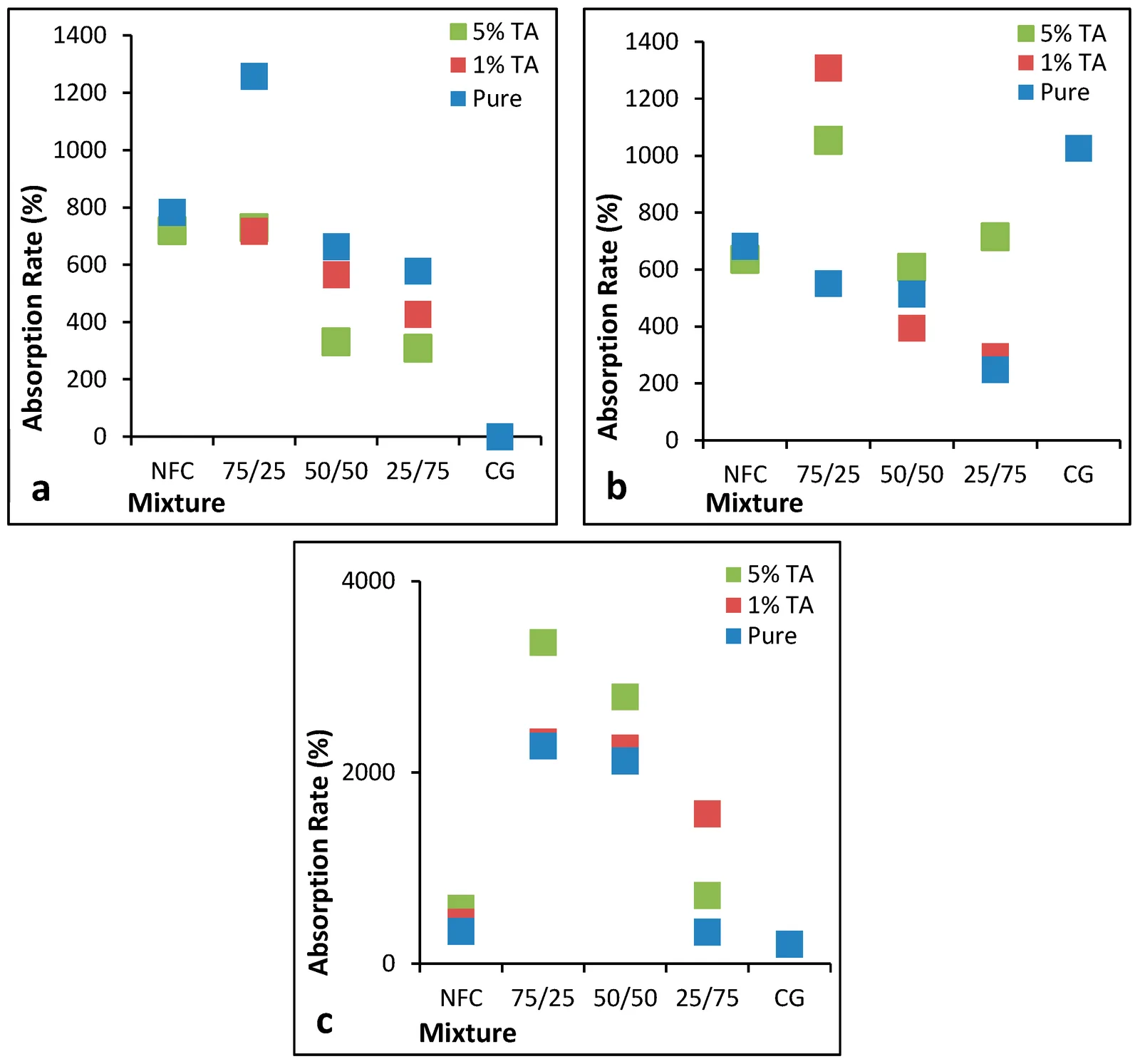
Water (**a**), PBS (**b**), and oil (**c**) absorption performances of aerogels.

Figure 2a–c illustrates the outcomes concerning the absorption of water, PBS, and oil by aerogels. The absorption performance figures for all three liquids indicate that the aerogels demonstrate superior absorption properties. Examination of the water absorption performance of the aerogels (Figure 2a) reveals that the most effective performance was found to be 1257% in aerogels formed with a 75/25 mixing ratio, whilst the lowest performance was 308.69% in aerogels formulated with a 25/75/5C ratio. Owing to its hydrophilic properties, 100% CG-based aerogels dissolved fast in water, rendering absorption levels indeterminable.

Figure 2b presents the PBS absorption findings for the aerogels. The different salt structures in the PBS solution exhibited varying absorption tendencies in the aerogels, dependent upon their molecular weights. The maximum absorption rate was recorded in the 75/25/1C aerogel, at 1308%. The PBS absorption characteristics of both TA-free and 1% TA-added aerogels exhibited almost analogous patterns to their water absorption capabilities. Enhancing the amount of CG included in the NFC structure led to a reduction in PBS absorption values. Nonetheless, higher PBS absorption values were identified in aerogels formulated with a 5% TA addition. The increased absorption rate of salt solutions for aerogels containing 5% TA additive, despite slightly reduced porosity, can be attributed to the effective and rapid interaction between the cationic ions (Na^+^ and K^+^) in the salt solution and the high number of anionic OH^−^ and COO^−^ groups within the TA structure.

Figure 2c illustrates the oil absorption findings of the aerogels, with the maximum oil absorption values recorded at the mixing ratios of 75/25 (3357%) and 50/50 (2789%) of the aerogels containing a 5% TA additive. The figure clearly illustrates that aerogels with a significant amount of TA (5%) exhibit greater oil absorption values than the other two fractions (1% TA added and pure aerogels). The enhancement of oil absorption in aerogel structures can be attributed to the incorporation of crosslinking TA, elucidated by the structural stabilization and optimization conferred by the optimal quantity of TA inside the porous framework. TA, characterized by its higher concentration of -OH groups and polyphenolic attributes, functions as an effective crosslinker by establishing multiple hydrogen bonds with both NFC and CG. The TA’s behavior maintained the stability of the three-dimensional network and prevented potential cell collapse, facilitating the diffusion of oil, which includes growth molecules, into the porous structure, hence yielding elevated absorption values [68]. The high porosity and low density of the aerogels are expected to physically absorb the oil in the pore network via capillary action, a mechanism often reported for cellulose-based aerogel oil sorbents [69]. However, there is the possibility of polymer leaching into the oil phase, which was not directly examined in this study. Given the inherently polar/hydrophilic character of both NFC and CG, their solubility in the non-polar oil phase would be expected to be low [70]. This assumption, however, was not experimentally verified (e.g., via compositional analysis of the absorbed oil phase), and is another limitation of the present study.

### 3.3. Dye Penetration Ratio and WVTR

**Figure 3:**
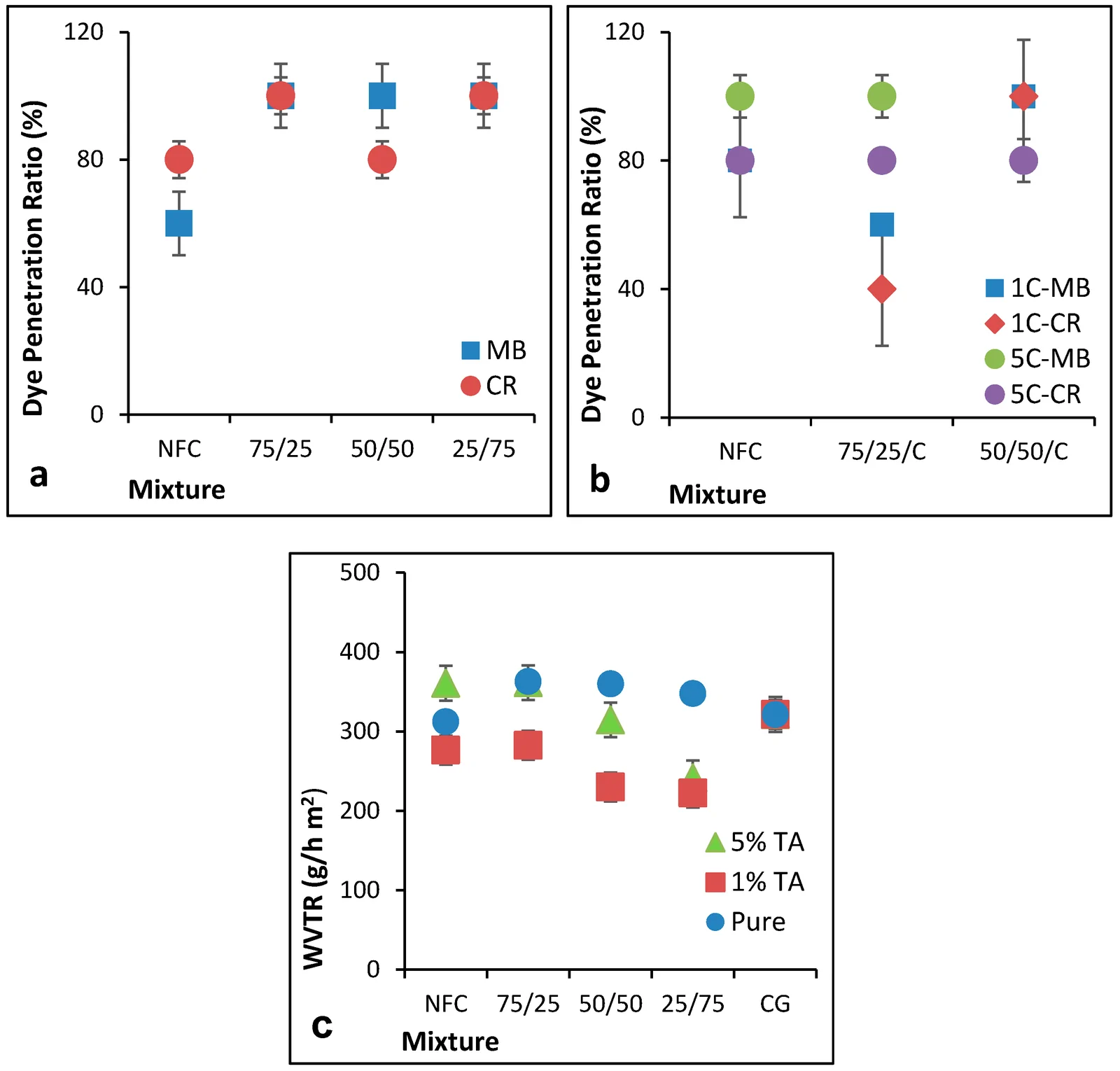
Dye penetration ratio (**a**,**b**) and WVTR performances (**c**) of aerogels.

Figure 3a–c presents the dye penetration test and WVTR outcomes of TA-free and TA-added aerogels. In porous materials, surface structures and pore-size distributions are critical factors in dye penetration and WVTR. Figure 3a illustrates that the cross-sectional penetration ratio of both dyes (MB and CR) from the aerogel surface to the bottom exceeded 50%. The penetration rate was calculated by dividing the distance traversed by the dye at a certain time by the total cross-sectional length. The minimum dye penetration ratio recorded was 60% for pure NFC with MB dye, whereas aerogels with 75/25 and 25/75 mixing ratios exhibited 100% penetration for both dyes. The complete dye penetration ratio signifies that the dye released from the aerogel’s surface reached the bottom within the set timeframe, hence supporting the material’s high porosity.

Dye penetration studies conducted using TA-added aerogels revealed varying penetration ratios among samples produced at varied mixing ratios, with findings illustrated in Figure 3b. The most elevated penetration ratios were recorded in samples formulated with NFC/5C and a 75/25/5C. The minimum penetration ratio was founded at 40% for CR diffusion in aerogels containing a 1% TA additive. Aerogels prepared with a 75/25 mixing ratio exhibited varying dye penetration ratios based on varied TA addition rates; conversely, with a 50/50 mixing ratio, a 5% TA addition adversely impacted the penetration ratio, despite comparable porosity levels. Despite comparable porosity values, the different dye penetration ratios of TA-added and TA-free aerogels can be attributed to their distinct structural compositions. Although production conditions have stabilized, consistently producing porous composite materials such as foams, aerogels, and films with identical micromorphological structures remains unfeasible; nonetheless, analogous morphologies can be achieved. This scenario may result in varying properties in aerogel structures, including liquid absorption and dye penetration.

Figure 3c shows the WVTR performances of all aerogels. Analysis of the data reveals that the highest WVTR values are predominantly found in pure NFC-CG-based aerogels. In contrast, a comparison between crosslinker-added and non-additive aerogels indicates that NFC-CG/1C-based aerogels exhibit lower WVTR values. Examination of 100% NFC-based aerogels (pure NFC, 1% TA, and 5% TA) reveals that the maximum WVTR value, 360.7 g/h m^2^, was associated with the addition of 5% TA, whilst the lowest value, 276.6 g/h m^2^, was observed in aerogels containing 1% TA. Pure NFC (without TA) aerogels exhibited a WVTR value of 312.45 g/h m^2^. The WVTR value of 100% CG-based aerogels was determined to be 321.30 g/h m^2^. The addition of a small quantity of CG into the NFC structure raised the WVTR values relative to aerogels composed entirely of NFC; however, these values subsequently diminished with an increase in CG concentration.

### 3.4. XRD

**Figure 4:**
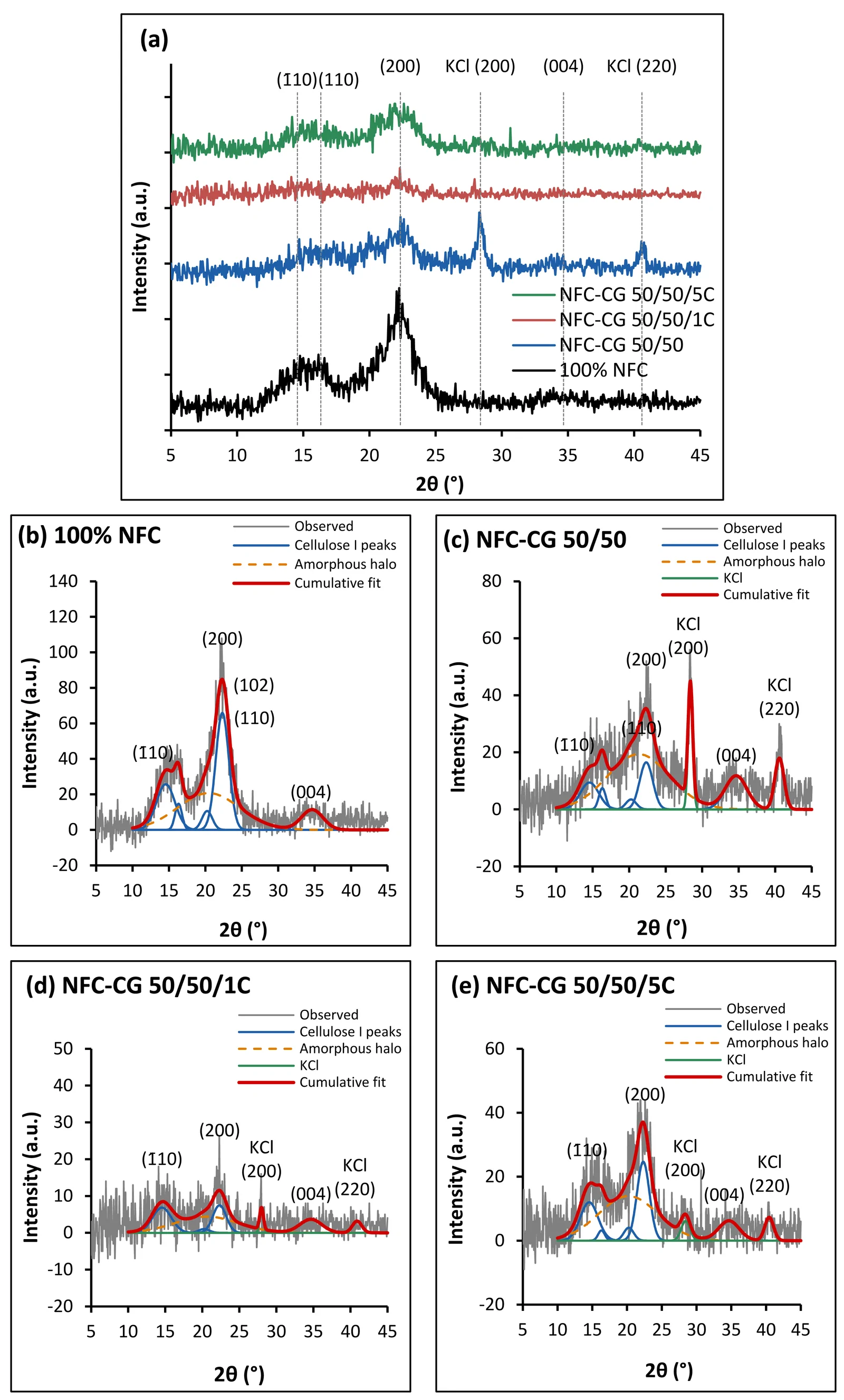
(**a**) XRD patterns of 100% NFC, NFC-CG 50/50, NFC-CG 50/50/1C, and NFC-CG 50/50/5C aerogels (vertically offset for clarity), with the cellulose Iβ and KCl reflections indexed. (**b**–**e**) Peak deconvolution of each pattern into cellulose Iβ crystalline peaks, a non-crystalline halo, and KCl peaks (fitted parameters in Table S1).

Figure 4 presents the XRD patterns of the selected aerogels, including 100% NFC, NFC-CG 50/50, NFC-CG 50/50/1C, and NFC-CG 50/50/5C. The 100% NFC aerogel exhibits reflections of cellulose Iβ at 2θ ≈ 14.6° (110), 16.3° (110), 20.3° (102)/(012), 22.3° (200), and 34.7° (004), while the non-crystalline fraction appears as a broad halo centered at approximately 20° (Figure 4a). The deconvoluted patterns are shown in Figure 4b–e and the fitted parameters are given in Table S1. The incorporation of CG into the structure results in diminished intensities of the cellulose reflections, whereas the (200) reflection remains at ~22.3° in all aerogels and no new cellulose reflections appear after the addition of CG or TA, indicating that the cellulose I lattice is preserved. The sharp reflections at 28.4° and 40.6°, observed only in the CG-containing aerogels, correspond to the (200) and (220) planes of KCl, which most likely originate from the commercial κ-carrageenan, in which potassium salts are commonly present.

In particular, the intensity of the main cellulose I diffraction maximum decreased in the NFC-CG 50/50 aerogel compared with the 100% NFC aerogel. This change is consistent with the contribution of the predominantly amorphous CG phase and the interactions between CG and the NFC matrix. Therefore, the reduction in the calculated Segal index should be interpreted as a decrease in the apparent/relative cellulose-related crystalline contribution rather than as a direct measurement of the total crystalline fraction of the NFC-CG composite.

The Segal CI of the aerogels were 49.7, 39.2, 38.5, and 38.0% for 100% NFC, NFC-CG 50/50, NFC-CG 50/50/1C, and NFC-CG 50/50/5C, respectively. The CI obtained from the peak deconvolution (CI-d) likewise decreased from 58.5% for 100% NFC to 37.9% for NFC-CG 50/50. The CI-d values of the TA-containing aerogels (53.7% for 50/50/1C and 46.3% for 50/50/5C) should be regarded as indicative because of the low diffracted intensity of these samples; the differences among the CG-containing aerogels are within the uncertainty of the method. The reduction from 49.7% for the NFC aerogel to 39.2% for the NFC-CG 50/50 aerogel indicates a lower relative contribution of the cellulose crystalline structure after incorporation of CG. This behavior may be associated with the amorphous nature of CG and its interactions with the NFC network, which can modify the packing and ordering of the cellulose fibrils. The addition of TA resulted in only a relatively small further decrease in the calculated CI, from 39.2% for NFC-CG 50/50 to 38.5% and 38.0% for the corresponding 1% and 5% TA-containing aerogels, respectively. These changes should not be interpreted as evidence that TA directly destroyed the cellulose crystalline domains. Rather, they suggest that the incorporation of TA modified the molecular organization and intermolecular interactions within the NFC-CG network, with the observed changes being reflected in the relative cellulose-related diffraction profile. The absence of new reflections and of a significant change in crystallinity after the addition of TA indicates that TA did not modify the inherent crystalline structure of cellulose I, but instead mostly engaged with the amorphous areas of the polymer matrix. The XRD results therefore support the interpretation that CG and TA primarily modify the organization and relative contribution of the cellulose-rich phase rather than produce a new crystalline structure. Ganesan and Ratke (2014) [71] performed XRD measurements in their investigation of a CG-based aerogel, observing the absence of unique peak patterns, which they attributed to the orientation arrangement of helical structures during synthesis. As a result, only a slight decrease in the Segal CI was noted, without the emergence of new crystalline diffraction peaks. Figure 4b distinctly illustrates the crystalline and non-crystalline contributions in the 100% NFC aerogel (Segal CI 49.7%). The value was established as 50.2 in the research performed by Kurtuluş et al. (2023) [72], whereas the CI value of the MBP used for NFC manufacture was ascertained to be 75.8. Likewise, it is evident that TEMPO oxidation, succeeded by homogenization, enhances the development of amorphous regions by affecting the crystalline structure inside the NFC structure.

### 3.5. FTIR

**Figure 5:**
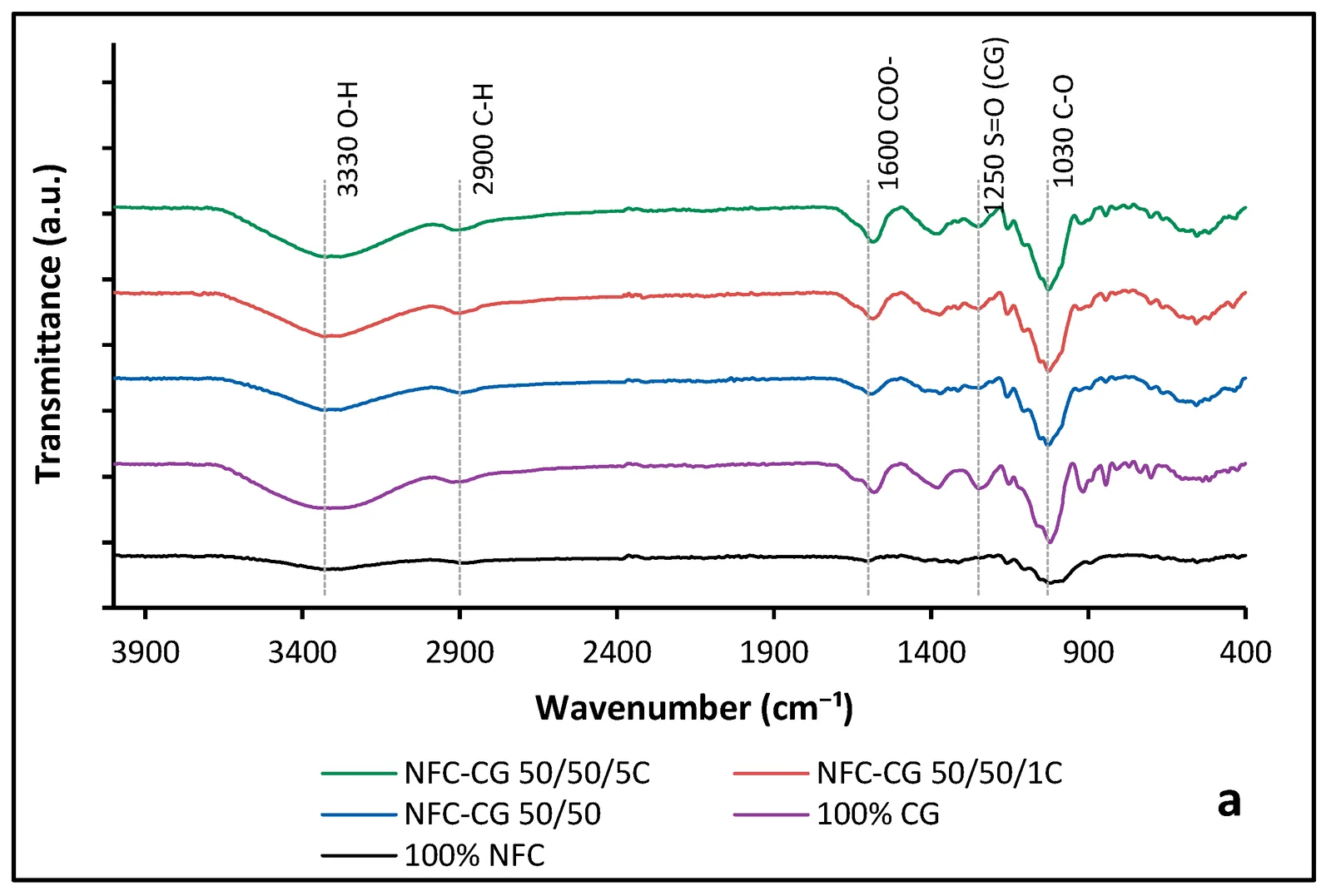

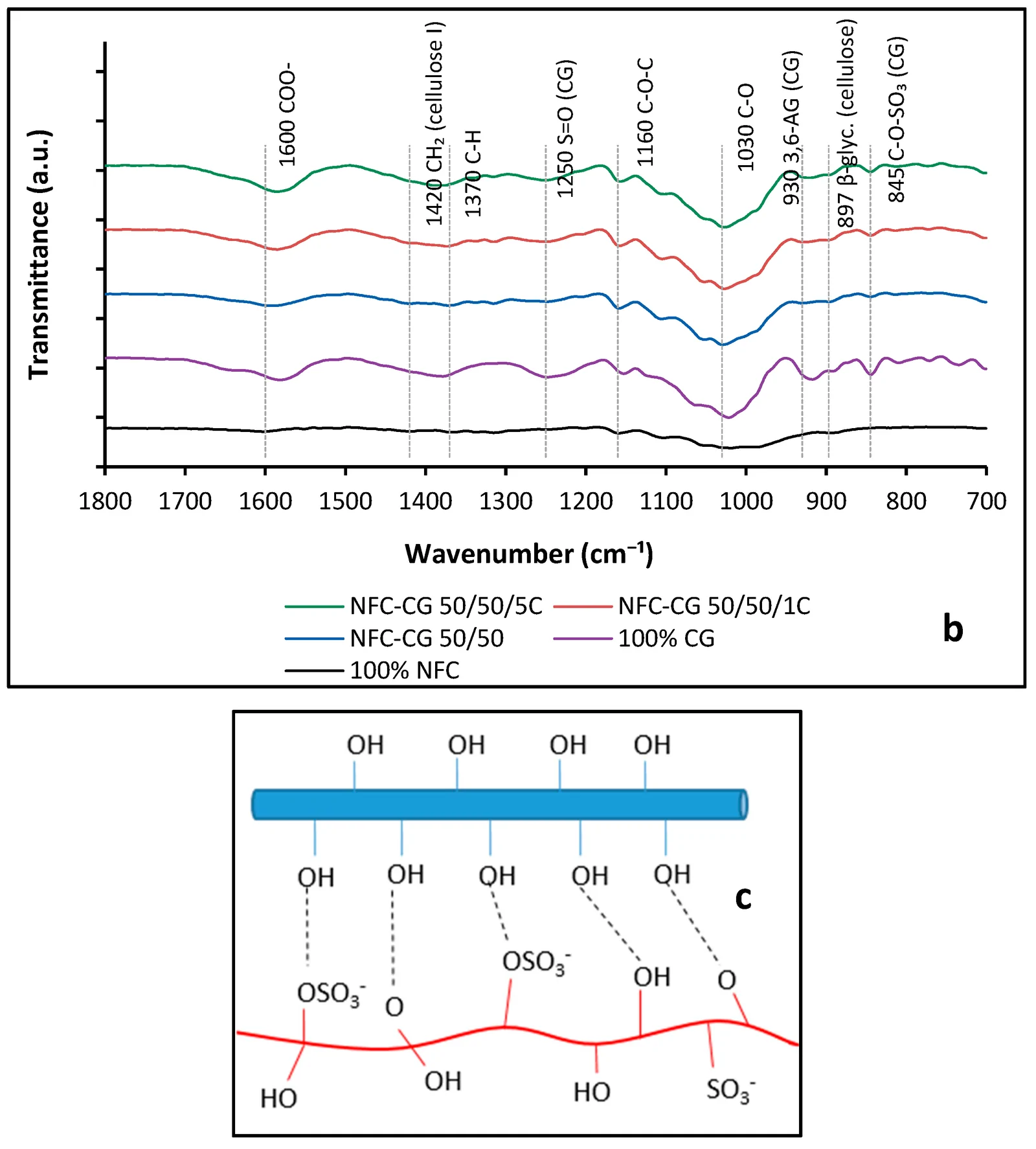
FTIR spectra of the aerogels: (**a**) 4000–400 cm^−1^ and (**b**) fingerprint region 1800–700 cm^−1^ with band assignments; (**c**) interaction scheme of NFC and CG.

Figure 5a–c illustrates the FTIR spectra of the aerogels and provides a schematic view of the chemical interactions between the polymers. The FTIR spectrum of the 100% NFC-based aerogel reveals that the peak at 1420 cm^−1^ corresponds to the CH_2_ scissoring (bending) vibration of cellulose, whereas the peak at 1030 cm^−1^ indicates the skeletal vibrational deformation of the C-O-C pyranose ring [73,74]. The 1367 cm^−1^ C-H deformation vibration corresponds to the 1314 cm^−1^ H-C-H fluctuating vibration, while the peaks at 1202 cm^−1^, 1159 cm^−1^, 1052 cm^−1^, and 895 cm^−1^ are attributed to C-O-H plane deformation, C-O-C asymmetric bridge stress of β-glycosidic bonds, C-O-C pyranose ring skeleton vibration, and C_1_-O-C_4_ deformation of β-glycosidic bonds, respectively [74,75]. The detection of carboxyl groups in the NFC structure resulting from TEMPO oxidation occurred at a peak of 1727 cm^−1^ [76]. The FTIR spectrum of the CG-based aerogel reveals peaks indicative of the unique structure of CG. The peaks at 3311 cm^−1^ and 2920 cm^−1^ correspond to the OH stretch and the CH stretch, respectively. The peaks at 1247 cm^−1^ and 1152 cm^−1^ correspond to the sulfate stretching of S-O and the stretching of C-O-H, respectively. The 1036 cm^−1^ peak observed at 1021 cm^−1^ is attributed to the C-O-C structure of 3,6-anhydrogalactose, whereas the 917 cm^−1^ peak is ascribed to the C-O-SO_3_ stretching vibration. Additionally, the peaks at 844 cm^−1^ and 700 cm^−1^ observed in the CG structure indicate the presence of sulfate inside the C4 galactose structure [77,78,79,80,81].

Upon examination of the FTIR spectra of aerogels formed with a 50/50 mixing ratio, both with and without crosslinking additives, broadly analogous peak structures were noted, albeit with some variations in peak intensities. In the pure CG structure, the 3311 cm^−1^ peak was displaced to 3326 cm^−1^; however, the strength of the 1581 cm^−1^ peak diminished. The 1378 cm^−1^ peak migrated to 1372 cm^−1^, and the 1247 cm^−1^ peak shifted to 1248 cm^−1^, indicating a reduced signal intensity. In the pure CG structure, the C-O-SO_3_ tension peak, initially at 917 cm^−1^, migrated to 930 cm^−1^ and was observed at a diminished intensity alongside the 844 cm^−1^ peak. The alterations in peak intensities indicate the existence of chemical interactions among the reactive groups. In the blends, the carboxylate band of NFC at ~1600 cm^−1^ overlaps with the 1581 cm^−1^ band of CG; therefore, a possible interaction of the NFC carboxylate groups with the –OH and –O-SO_3_ groups of CG cannot be resolved from this region alone. The characteristic cellulose bands at approximately 1430 cm^−1^ and 1370 cm^−1^, associated with CH_2_ bending and C–H deformation vibrations, were preserved in all aerogels. Similarly, the pronounced absorption seen between 1160 and 1030 cm^−1^, attributed to C–O–C and C–O stretching vibrations of β-(1-4)-glycosidic bonds, confirms that the cellulose framework stays unaltered following composite fabrication. The presence of CG is indicated by the distinctive sulfate-associated absorption bands in the fingerprint region. The spectral band observed at about 1220–1260 cm^−1^ is associated with the stretching vibration of sulfate ester (S=O) moieties, whilst the absorption peaks at approximately 930 cm^−1^ and 845 cm^−1^ are linked to 3,6-anhydrogalactose and galactose-4-sulfate units, respectively. The distinctive peaks are strengthened in the aerogels, confirming the effective incorporation of CG into the NFC framework. Furthermore, upon analyzing the FTIR spectra of TA-added aerogels, it is evident that, despite exhibiting similar FTIR patterns to aerogels produced without crosslinking agents at a 50/50 mixing ratio, there is a notable increase in peak intensities. This observation contrasts with the previously noted changes, and these peaks correspond to the structural characteristic peaks of CG. This signifies that, upon the incorporation of TA, this chemical predominantly interacts with the –OH and carboxylate (–COO^−^) groups present in the NFC structure. Significantly, no new absorption bands are seen following crosslinking, indicating that this procedure primarily improves intermolecular contacts via hydrogen bonding rather than producing novel covalent functional groups identifiable by FTIR.

The FTIR results are consistent with the XRD analysis (Table S2). The LOI was highest for 100% NFC (1.66) and decreased to 1.40 for NFC-CG 50/50 and to 1.12–1.27 for the TA-containing aerogels. Since CG also absorbs near 1370–1380, 2900–2920, and 900–930 cm^−1^, the ratios of the blends were recalculated after subtraction of the scaled CG spectrum, giving 1.28 (50/50), 0.94 (50/50/1C), and 0.98 (50/50/5C). Both techniques therefore indicate that CG dilutes the crystalline cellulose phase and increases the disordered fraction, while the positions of the cellulose bands (1160, 1030, and 897 cm^−1^) and of the cellulose reflections remain unchanged. TA produced neither new reflections nor new absorption bands (e.g., no ester C=O band near 1710–1720 cm^−1^), indicating that it interacts mainly through hydrogen bonding in the disordered regions. The KCl reflections observed by XRD have no counterpart in the IR spectra, as KCl has no IR-active vibrations in the measured range.

### 3.6. Bacterial Resistance

**Figure 6:**
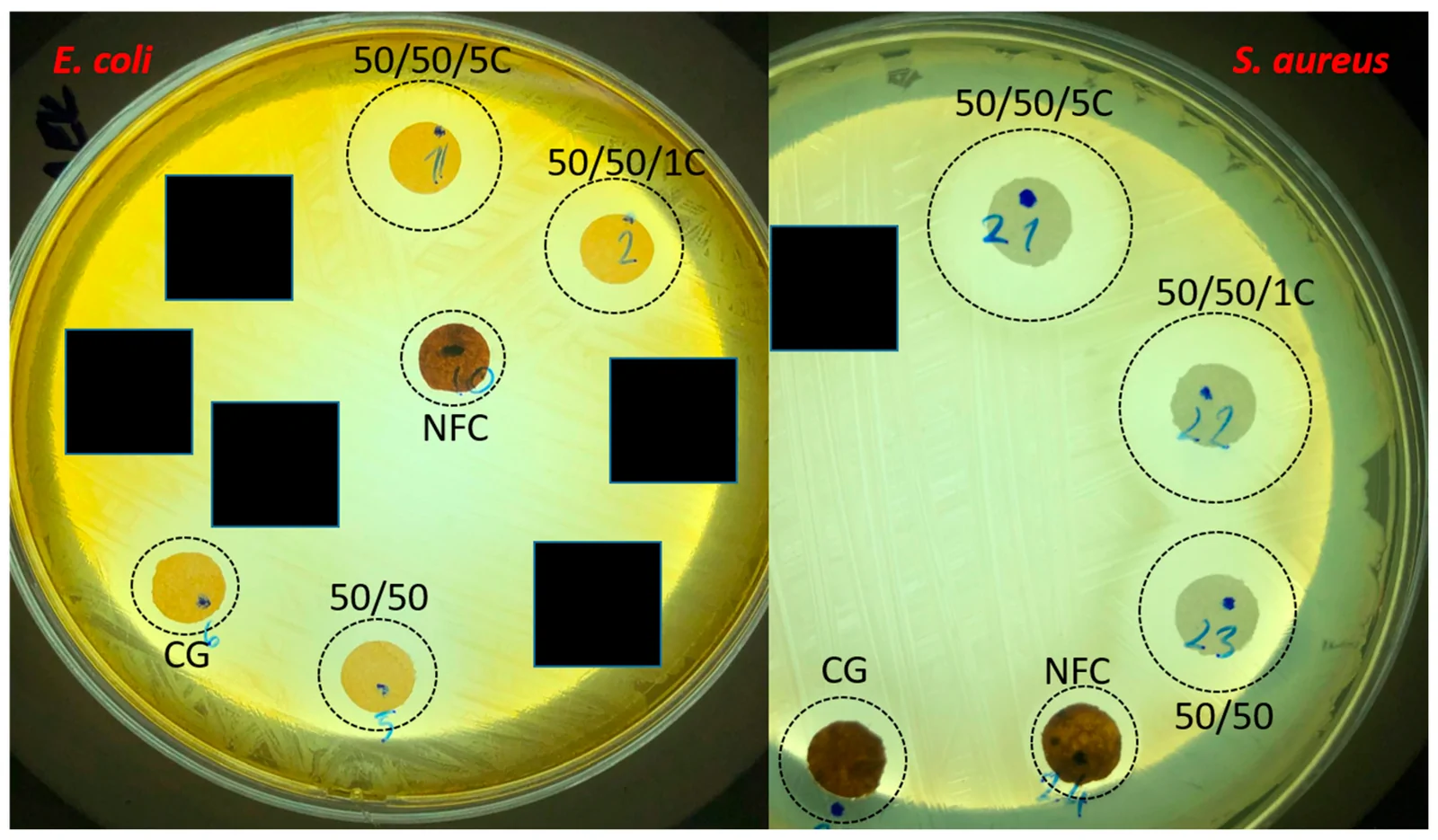
Inhibition zones of aerogels after antibacterial activity test.

The antibacterial efficacy of aerogels was assessed using the disk diffusion method. Bacteria cultivated in SS (*E. coli*)- and BHA (*S. aureus*)-enriched media were applied to agar media using the swab method, after which samples cut into 1 cm diameter pieces were placed in contact with the bacterial medium. The culture medium was incubated at 28 °C for 24 h, after which inhibition zones associated with bacterial growth were assessed on agar medium. Table 2 presents the measurements of the inhibition zones (Figure 6) for the aerogels, indicating that pure NFC-based aerogels showed only a limited antibacterial effect. The larger inhibition zones observed for *S. aureus* compared to *E. coli* in the pure NFC-based aerogels (Table 2) are attributed to the reduced net negative surface charges of Gram-positive bacteria. This phenomenon in oxidized nanocellulose is explained by electrostatic repulsion between anionic carboxylate groups and the negatively charged bacterial surface [82]. It is evident that all aerogels prepared with pure CG and an NFC-CG 50/50 mixing ratio demonstrate resistance to both bacteria. The table indicates that the antibacterial activity of aerogels synthesized with a crosslinking additive surpasses that of those synthesized without such an additive. This circumstance presumably arises from the synergistic effects of CG and NFC, both of which possess antibacterial capabilities, along with the contribution of TA, which also exhibits antibacterial efficacy. Due to the presence of phenolic hydroxyl groups in its structure, TA demonstrates a range of biofunctional properties, including antienzymatic, antioxidant, antimutagenic, antitumor, antiviral, anticarcinogenic, and hemostatic effects, and exhibits antibacterial activity against various bacterial species such as *S. aureus*, *E. coli*, and *H. pylori* [83,84,85]. Composite hydrogels based on NFC-CG, boosted with antibiotics, were examined for potential uses in periodontitis, and their antibacterial efficacy was assessed against *Streptococcus mutans*, *Porphyromonas gingivalis*, *Fusobacterium nucleatum*, and additional bacterial strains. The research indicated that NFC-CG-based hydrogels lacked resistance to *Streptococcus mutans* and *Fusobacterium nucleatum*, although resistance was shown when antibiotics were incorporated into their composition. NFC-CG-based hydrogels exhibited antibacterial resistance against two more bacterial species in the absence of antibiotics [86].

### 3.7. SEM (Scanning Electron Microscopy) Images

**Figure 7:**
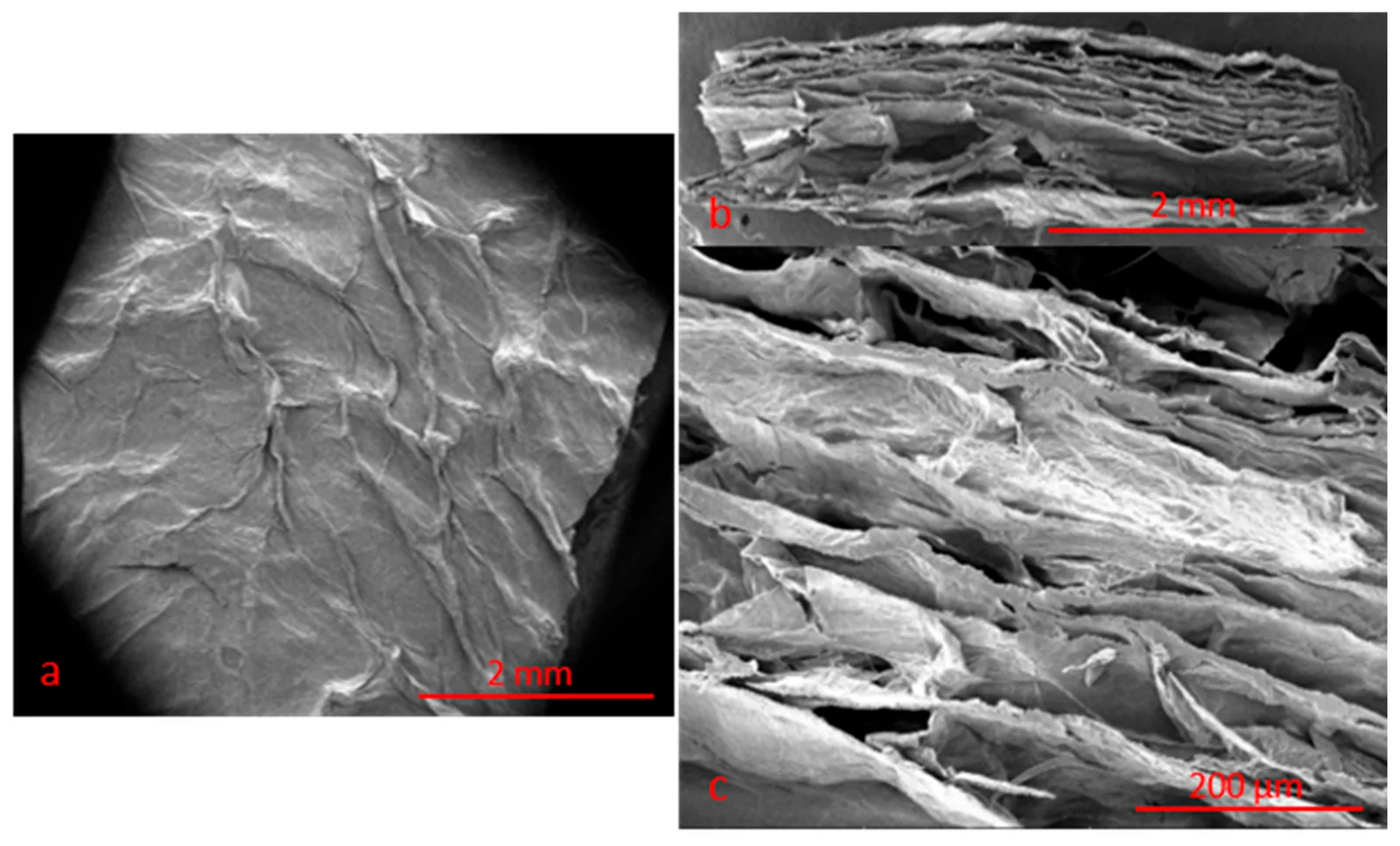
SEM images of 100% NFC: ((**a**) surface; (**b**,**c**) cross-section).

Figure 7 presents SEM images of aerogels with 100% NFC, 50/50, and 50/50/5C compositions. Upon analyzing Figure 7a–c, it is evident that the NFC structure shows a fibrous formation on its surface (Figure 7a). The fibrous multilayered structure, coupled with the parallel alignment of the fibers, is distinctly observable on the surface. Analysis of the cross-sectional structure (Figure 7b,c) indicates that the nanofiber layers are organized in contact with one another, with substantial pores present between the layers. The image supports the elevated porosity values observed in the porosity assessments of 100% NFC-based aerogels.

**Figure 8:**
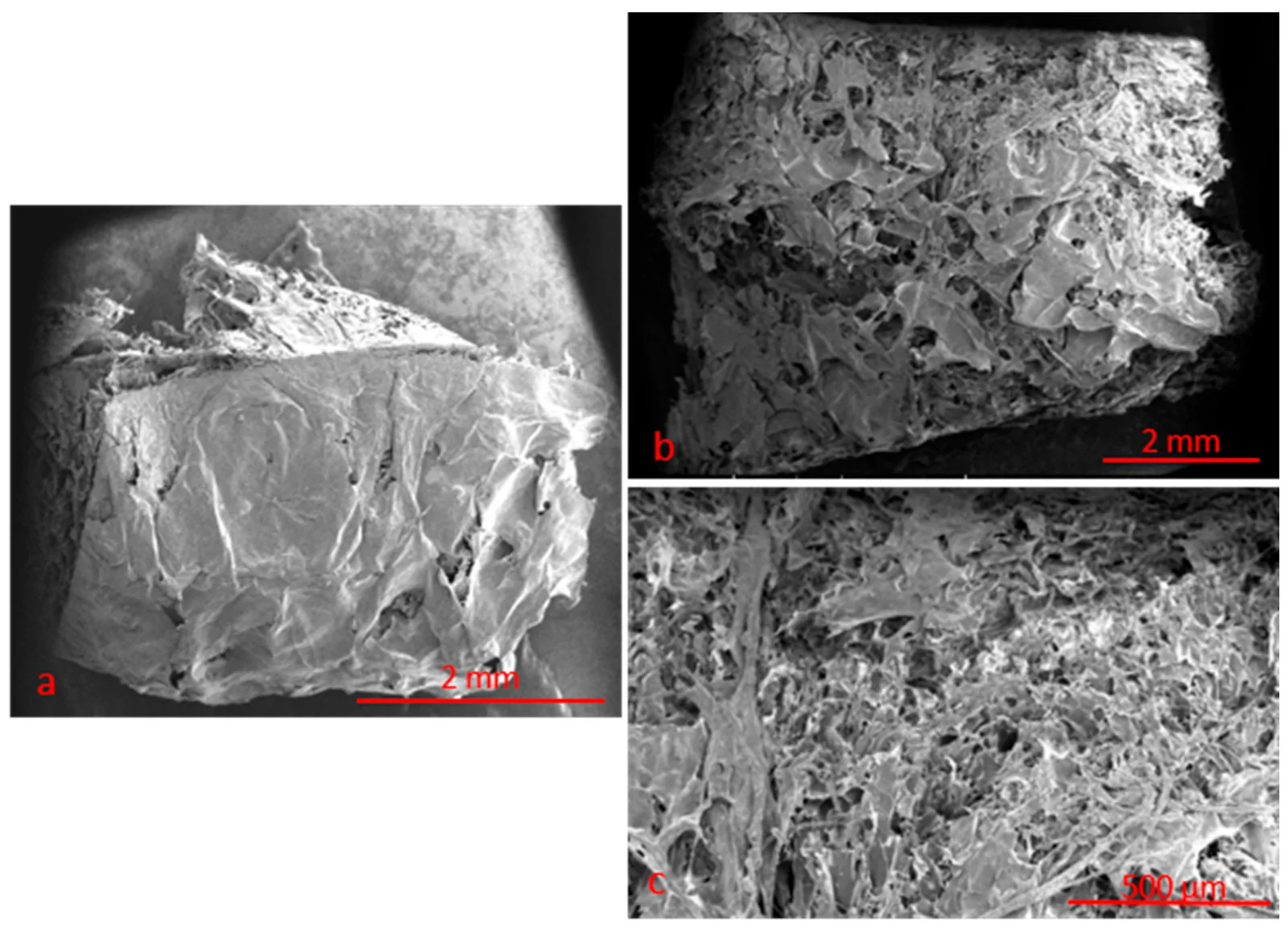
SEM images of NFC-CG 50/50: ((**a**) surface; (**b**,**c**) cross-section).

Upon examining the morphology of the aerogel produced with a 50/50 mixing ratio without crosslinking additives, it can be observed that the surface structure (Figure 8a) closely resembles that of 100% NFC-based aerogels, with nanofibers constituting the surface structure both individually and in aggregations. Nonetheless, in contrast to entirely NFC-based aerogels, a limited number of pores are discernible on the surface structure. The incorporation of CG into the NFC framework is believed to contribute to the development of this uniform structure on the surface. Upon examination of the cross-sectional structure, a very porous network has emerged, markedly distinct from the 100% NFC structure. Aerogels produced with a 50/50 mixing ratio display a distinct morphology of a porous structure in their cross-sectional regions compared to 100% NFC-based aerogels. The layered configuration present in 100% NFC-based aerogels is substituted with a denser, more abundant pore structure with a 50/50 mixing ratio. The sublimation process during the lyophilization phase of aerogel manufacture is considered a crucial aspect in the development of its distributed network structure. SEM images reveal the most pronounced quantities of CG additive and crosslinking additive in samples based on a 50/50/5C composition (Figure 9a–c). Upon examination of the figure, it is noted that an increase in the concentration of CG additive and crosslinking agent results in a notably dense surface morphology and cross-sectional structure of the aerogels. This scenario suggests that a high concentration of TA additive causes the polymeric chains to aggregate, leading to a deterioration in the porosity structure.

SEM pictures of aerogels, when analyzed with porosity and specific surface area metrics, yield complementary findings. The maximum porosity (99.63%) and surface area (303.12 m^2^/g) values were recorded for the 100% NFC-based aerogels, with extensive and abundant pores in the cross-sectional structures evident in the SEM images corroborating these findings. In crosslinked aerogels with a 50/50 mixing ratio, SEM images revealed a denser surface structure and cross-sectional topologies characterized by smaller pores, supported by diminished porosity and specific surface area values relative to 100% NFC. Upon the incorporation of 5% TA, the maximum concentration of crosslinking additive, the aerogels exhibit a highly dense and compact shape with regard to both surface and cross-sectional structures. The interactions of TA with both NFC and CG caused the polymer chains to aggregate, leading to reduced porosity and specific surface area measurements.

## 4. Conclusions

TEMPO-oxidized NFC-CG-based aerogels were produced by means of a high-pressure homogenizator in various mixing ratios (100% NFC, 75–25% NFC-CG, 50–50% NFC-CG, 25–75% NFC-CG, and 100% CG), both with and without a crosslinking agent (TA). The density–porosity, BET surface area, liquid absorption capacity, dye penetration, WVT, chemical structural alterations (FTIR), crystallinity degree (XRD), and antibacterial characteristics of the aerogels were analyzed, accompanied by an examination of their micromorphological structures via SEM imaging. Aerogels show densities ranging from 0.0154 to 0.0267 g/cm^3^, porosities between 97.91% and 99.63%, and specific surface areas of 128 to 303 m^2^/g, as indicated by the findings. All aerogels demonstrated exceptionally high water and PBS absorption rates, with WVT values ranging from 222 to 363 g/h m^2^. The incorporation of CG resulted in a decrease in the apparent cellulose-related CI, indicating a lower relative contribution of the ordered cellulose phase in the composite structure. Nonetheless, upon evaluation for bacterial resistance, aerogels demonstrated efficacy against both species, exhibiting a superior effectiveness against Gram-positive *S. aureus*. The findings suggest that aerogels may serve as appropriate materials not only for active packaging and filtering applications but also for biomedical uses, due to their remarkable liquid absorption properties, significant porosity, extensive surface area, and antibacterial efficacy.

## Figures and Tables

**Table 1:** Density, porosity, and surface area values of aerogels.

Sample	Density (g/cm^3^)	Porosity (%)	BET Surface Area (m^2^/g)
NFC	0.0223	99.63	303.12
CG	0.0236	98.77	128.25
NFC-CG 75/25	0.0155	99.52	285.03
NFC-CG 50/50	0.0169	99.41	255.28
NFC-CG 25/75	0.0255	99.21	251.02
NFC-1C	0.0258	99.58	301.03
NFC-CG 75/25/1C	0.0154	99.11	266.85
NFC-CG 50/50/1C	0.0156	98.88	231.23
NFC-CG 25/75/1C	0.0194	98.7	229.21
NFC-5C	0.0267	98.01	292.77
NFC-CG 75/25/5C	0.0154	98.51	248.45
NFC-CG 50/50/5C	0.0195	98.23	222.56
NFC-CG 25/75/5C	0.0262	97.91	209.12

**Table 2:** Inhibition zones of different aerogels versus *S. aureus* and *E. coli*.

Sample	*S. aureus* (mm)	*E. coli* (mm)
NFC	2.55	2.28
CG	3.02	2.33
NFC-CG 50/50	6.33	6.02
NFC-CG 50/50/1C	13.22	11.36
NFC-CG 50/50/5C	16.11	13.88

## Data Availability

The data presented in this study are available upon request from the corresponding author.

## Supplementary Materials

The following supporting information can be downloaded at: https://www.mdpi.com/article/10.3390/polym18202464/s1, Table S1: Parameters of the XRD peak deconvolution (Gaussian components. 2θ = 10–45°). In the CG-containing aerogels the positions and widths of the cellulose Iβ peaks were fixed to the values of 100% NFC and only their intensities were refined. CI-d = sum of the areas of the cellulose Iβ peaks/(sum of the areas of the cellulose Iβ peaks + area of the non-crystalline halo) × 100; KCl peaks are excluded; Table S2: Comparison of the XRD crystallinity indices with the IR crystallinity ratios (LOI = A1430/A897. TCI = A1372/A2900). CG-corrected values were calculated after subtraction of the pure CG spectrum scaled on the 845 cm^−1^ band. The ratios are used to compare trends between samples. not as absolute crystallinity values.

## Abbreviations

The following abbreviations are used in this manuscript:

NFC	Nanofibrillated cellulose
CG	Carrageenan
TA	Tannic acid
BET	Brunauer–Emmett–Teller
PBS	Phosphate-buffered saline
MB	Methylene blue
CR	Congo red
WVTR	Water vapor transmission rate
FTIR	Fourier transform infrared spectroscopy
XRD	X-ray diffraction
SEM	Scanning electron microscopy
CI	Crystallinity Index
MBP	Magnesium bisulfite pulp
