# Supplementary material for: Nanofibril Cellulose–Carrageenan Aerogel Production and the Effect of Crosslinker Addition on Aerogel Performance
Source: Polymers (Basel). 2026 Oct 9;18(20):2464. doi: 10.3390/polym18202464 (PMC13708957; doi:10.3390/polym18202464)
Supplement: Supplementary file 1 [file polymers-18-02464-s001.zip › polymers-4521638-supplementary.pdf]

Supplementary File

Table S1. Parameters of the XRD peak deconvolution (Gaussian components. 2θ = 10–45°). In the CG-containing aerogels the positions and widths of the cellulose Iβ peaks were fixed to the values of 100% NFC and only their intensities were refined. CI-d = sum of the areas of the cellulose Iβ peaks / (sum of the areas of the cellulose Iβ peaks + area of the non-crystalline halo) × 100; KCl peaks are excluded

**100% NFC**				
**Component**	**Assignment**	**2θ (°)**	**FWHM (°)**	**Area (%)**
(1-10)	Cellulose Iβ (1̄10)	14.56	3.00	15.5
(110)	Cellulose Iβ (110)	16.32	1.12	3.3
(102)	Cellulose Iβ (102)/(012)	20.25	1.67	3.6
(200)	Cellulose Iβ (200)	22.33	2.12	28.1
(004)	Cellulose Iβ (004)	34.66	3.50	8.0
Amorphous	Non-crystalline halo (amorphous cellulose + CG)	20.46	10.00	41.6
Crystallinity from deconvolution. CI-d (%) = ΣA(cellulose I peaks) / [ΣA(cellulose I peaks) + A(amorphous)] × 100 (KCl peaks excluded)				58.45
R^2 (fit window)				0.90
RMSE (a.u.)				6.18

**NFC-CG 50/50**				
**Component**	**Assignment**	**2θ (°)**	**FWHM (°)**	**Area (%)**
(1-10)	Cellulose Iβ (1̄10)	14.56	3.00	7.7
(110)	Cellulose Iβ (110)	16.32	1.12	2.3
(102)	Cellulose Iβ (102)/(012)	20.25	1.67	1.6
(200)	Cellulose Iβ (200)	22.33	2.12	9.5
(004)	Cellulose Iβ (004)	34.66	3.50	11.1
Amorphous	Non-crystalline halo (amorphous cellulose + CG)	21.29	10.00	52.5
KCl (200)	KCl (200). sylvite	28.38	0.74	8.1
KCl (220)	KCl (220). sylvite	40.58	1.50	7.3
Crystallinity from deconvolution. CI-d (%) = ΣA(cellulose I peaks) / [ΣA(cellulose I peaks) + A(amorphous)] × 100 (KCl peaks excluded)				37.94
R^2 (fit window)				0.66
RMSE (a.u.)				5.88

**NFC-CG 50/50/1C**				
**Component**	**Assignment**	**2θ (°)**	**FWHM (°)**	**Area (%)**
(1-10)	Cellulose Iβ (1̄10)	14.56	3.00	19.9
(110)	Cellulose Iβ (110)	16.32	1.12	0.0
(102)	Cellulose Iβ (102)/(012)	20.25	1.67	1.7
(200)	Cellulose Iβ (200)	22.33	2.12	15.4
(004)	Cellulose Iβ (004)	34.66	3.50	12.4

NFC-CG 50/50/5C				
**Component**	**Assignment**	**2θ (°)**	**FWHM (°)**	**Area (%)**
(1-10)	Cellulose Iβ (1̄10)	14.56	3.00	12.9
(110)	Cellulose Iβ (110)	16.32	1.12	1.3
(102)	Cellulose Iβ (102)/(012)	20.25	1.67	2.4
(200)	Cellulose Iβ (200)	22.33	2.12	18.7
(004)	Cellulose Iβ (004)	34.66	3.50	7.7
Amorphous	Non-crystalline halo (amorphous cellulose + CG)	20.19	10.00	49.8
KCl (200)	KCl (200). sylvite	28.42	1.50	3.3
KCl (220)	KCl (220). sylvite	40.45	1.50	3.9
Crystallinity from deconvolution. CI-d (%) = ΣA(cellulose I peaks) / [ΣA(cellulose I peaks) + A(amorphous)] × 100 (KCl peaks excluded)				46.31
R^2 (fit window)				0.74
RMSE (a.u.)				4.75
Amorphous	Non-crystalline halo (amorphous cellulose + CG)	20.60	10.00	42.6
KCl (200)	KCl (200). sylvite	28.00	0.59	3.4
KCl (220)	KCl (220). sylvite	40.90	1.50	4.6
Crystallinity from deconvolution. CI-d (%) = ΣA(cellulose I peaks) / [ΣA(cellulose I peaks) + A(amorphous)] × 100 (KCl peaks excluded)				53.67
R^2 (fit window)				0.28
RMSE (a.u.)				3.33

Table S2. Comparison of the XRD crystallinity indices with the IR crystallinity ratios (LOI = A1430/A897. TCI = A1372/A2900). CG-corrected values were calculated after subtraction of the pure CG spectrum scaled on the 845 cm^{-1} band. The ratios are used to compare trends between samples. not as absolute crystallinity values

Sample	XRD CI-d (%) deconvolution	XRD CI (%) Segal (manuscript)	IR LOI raw	IR TCI raw	IR LOI CG-corrected	IR TCI CG-corrected
100% NFC	58.5	49.7	#AD?	#AD?	#AD?	#AD?
NFC-CG 50/50	37.9	39.2	#AD?	#AD?	#AD?	#AD?
NFC-CG 50/50/1C	53.7	38.5	#AD?	#AD?	#AD?	#AD?
NFC-CG 50/50/5C	46.3	38	#AD?	#AD?	#AD?	#AD?
